# Circular Approach to Composite Materials: Synthesis of Carbon Nanomaterials from Polymer Recycling Liquid By-Products

**DOI:** 10.3390/ma19061266

**Published:** 2026-03-23

**Authors:** Evangelos Tsimis, Stefania Termine, Maria Modestou, Aikaterini-Flora Trompeta, Szymon Sobek, Marcin Sajdak, Jakub Adamek, Sebastian Werle, Costas Charitidis

**Affiliations:** 1 Research Lab of Advanced, Composite, Nano-Materials and Nanotechnology (R-NanoLab), Materials Science and Engineering Department, School of Chemical Engineering, National Technical University of Athens, 9 Heroon Polytechniou, 15773 Athens, Greece; etsimis@chemeng.ntua.gr (E.T.); stermine@chemeng.ntua.gr (S.T.);; 2 Department of Heating, Ventilation and Dust Removal Technology, Faculty of Energy and Environmental Engineering, Silesian University of Technology, Stanisława Konarskiego 20, 44-100 Gliwice, Poland; szymon.sobek@polsl.pl; 3 Department of Air Protection, Faculty of Energy and Environmental Engineering, Silesian University of Technology, Stanisława Konarskiego 22B, 44-100 Gliwice, Poland; marcin.sajdak@polsl.pl; 4 Department of Organic Chemistry, Bioorganic Chemistry and Biotechnology, Faculty of Chemistry, Silesian University of Technology, B. Krzywoustego 4, 44-100 Gliwice, Poland; jakub.adamek@polsl.pl; 5 Department of Thermal Technology, Faculty of Energy and Environmental Engineering, Silesian University of Technology, Stanisława Konarskiego 22, 44-100 Gliwice, Poland; sebastian.werle@polsl.pl

**Keywords:** carbon nanotubes, chemical vapor deposition, circular economy, polymeric composites solvolysis, waste upcycling

## Abstract

The growing volume of fiber-reinforced polymer composite waste creates an urgent need for efficient recycling technologies. While solvolysis effectively breaks down thermoset matrices for fiber reinforcement recovery, the process generates hydrocarbon-rich liquid by-products that require further management. This study validates the use of these liquid recycling streams—derived from the solvolysis of unsaturated polyester and epoxy resins—as sustainable carbon precursors for the growth of carbon nanomaterials. Synthesis was performed via catalytic chemical vapor deposition (CVD) at 850 °C using iron nanoparticles impregnated on a zeolite substrate. Morphological analysis confirmed the production of one-dimensional nanostructures (carbon nanotubes/nanofibers), with average diameters below 100 nm. Raman spectroscopy revealed a high degree of graphitization, with I_D_/I_G_ ratios ranging from 0.25 to 0.58, which is comparable to structures synthesized from conventional precursors. Thermogravimetric analysis (TGA) demonstrated high thermal stability and carbon purity reaching up to 90.3%. These findings demonstrate a viable upcycling pathway that enhances the economic attractiveness of composite recycling by transforming waste into advanced nanomaterials.

## 1. Introduction

Fiber-reinforced polymer matrix composites are one of the most important and rapidly growing categories of modern materials, widely used in the aerospace, automotive, 

Marine, and wind energy sectors [1]. Consequently, the volume of composite waste is projected to rise significantly [2], creating an urgent need for efficient recycling technologies. This is particularly challenging for thermoset matrix composites; unlike thermoplastics, they cannot be melted and remolded, causing the majority of this waste to end up in landfills [3].

Chemical recycling overcomes the challenges of thermoset polymer recycling, by breaking down the polymer matrix of composite materials into monomers and oligomers. When this is done using solvents and catalysts, the process is referred to as solvolysis and aims to break down the polymer network to effectively recover the reinforcing fibers [4]. In polyesters this can be achieved through polyethylene glycol (PEG)/NaOH, applied during solvolysis, which promotes glycolysis reactions via transesterification, leading to the decomposition of the unsaturated polyester resin matrix. Under alkaline conditions, hydroxyl anions and PEG-derived alkoxide species act as nucleophiles and interact with the electrophilic carbonyl groups of ester linkages within the polymer network. This nucleophilic substitution results in ester bond cleavage, reduction in crosslink density, and the formation of PEG-ester and hydroxyl-terminated oligomers with increased solubility in polar solvents [5,6].

The by-products of the chemical recycling of polymers vary depending on the nature of the polymer and the recycling process used, but in all cases they consist of a number of different chemical compounds, monomers, oligomers, and other derivatives that can be used for energy recovery or the synthesis of new materials [7]. As an example, carboxylic acids and alcohols have been identified as by-products during the solvolysis of epoxy resin with polyethylene glycol and caustic soda [8].

Despite the technical feasibility of thermal and chemical recycling, industrial adoption remains limited. Facilities across Europe are currently insufficient to meet recycling demands [9]. The primary obstacle is economic viability; recycled products often struggle to compete with traditional petroleum-based materials in terms of cost and quality [10]. Therefore, further innovation is required to create high-value applications that enhance the economic attractiveness of these recycling methods.

Upcycling the by-products of thermal and chemical recycling into high-value materials offers a promising strategy for managing non-recyclable thermoset waste and improving the sector’s economic viability. Recent advancements illustrate this potential, as numerous studies demonstrate a successful synthesis of carbon nanotubes (CNTs) from plastic waste [11,12,13,14,15,16,17]. These findings underscore the flexibility of Chemical Vapor Deposition (CVD), which has successfully utilized diverse non-virgin precursors like biomass [18] and plastic residues. Given that liquid wastes from composite recycling share a similar chemical profile, being complex, carbon-rich mixtures of monomers, oligomers, and solvents [8], they represent promising candidates for this process. Furthermore, this approach contributes to circularity, as the introduction of carbon nanofillers into a polymer matrix significantly enhances its electrical and thermal conductivity by forming conductive networks [19], presenting a viable circular use case. Accordingly, the current investigation aims to validate the use of polymer recycling liquid waste as a carbon source for carbon nanomaterial growth through CVD and to address the practical challenges of integrating these materials into a CVD reactor.

## 2. Materials and Methods

### 2.1. Precursor Materials

Four different samples of wind turbine blades composite material recycling liquid by-products were collected for use as precursor compounds for the growth of CNTs through CVD. The samples include solvolysis by-products coming from the recycling of unsaturated polyester and epoxy resin composite reinforced with glass fibers. The exact conditions of the recycling solvolysis processes that were carried out are given in Table 1 and described below.

Specifically, the polyester-derived liquid waste (SB1) that was used in this study was sourced from the chemical-assisted solvolysis experiments conducted to depolymerize the resin matrix of glass fiber-reinforced polymer (GFRP) composite materials from wind turbine blades (A TEC, Goedersdorf, Austria). All solvolysis runs were performed at atmospheric pressure in a thermostatically regulated reactor (Anton Paar, Graz, Austria), with PEG as a solvent (TechLine, Athens, Greece) and reaction times ranging between 4 and 5.5 h, and NaOH (TechLine, Athens, Greece) catalyst charges between 0.1 g and 2.0 g per gram of initial resin content. Under optimal conditions (200 g PEG, 12.5 g NaOH, and 10 g of GFRP reacted for 5.5 h), the solvolysis process achieved approximately 80% decomposition of the unsaturated polyester resin, resulting in the recovery of glass fibers with minimal structural damage [20]. Following each run, the liquid fraction was separated from the solid residue through filtration and collected in pre-labeled, inert containers for subsequent use.

Epoxy-derived liquid solvolysis by-products were also used in this study. The EoL composite came from wind turbine blade (WTB) samples (Ventos Metodicos, Troporiz, Portugal) in the form of pre-cut 25 × 30 cm segments. For the solvolysis processing, the samples were further cut into 0.5 cm and milled into final particle size of 0.5–1.5 cm chips using a shredder mill. The solvolysis experiments of the studied WTB samples were carried out using 3 L batch glass reactors coupled with dedicated heating spiral with a closed-loop system involving spiral cooler to condensate evaporating solvent back to the reaction zone. The following chemicals have been used: ethylene glycol (e.g., molecular weight of 62.07 kg/kmol, Merck, Darmstadt, Germany),1-methyl-2-pyrrolidinone (NMP; 99.13 kg/kmol, Merck, Darmstadt, Germany), and 1,5,7-Triazabicyklo[4.4.0]dek-5-en (TBD; 139.20 kg/kmol, Merck, Darmstadt, Germany). The solvolysis process parameters were adapted from a previous study of Muzyka et al. [21] and aimed at process optimization for the GFRP sample, i.e., process temperature T = 190 °C, processing time t = 3 h. The solvent used was EG-NMP (1:1 mol) solution, with an addition of 0.025 mol of TBD. The WTB sample mass for a single experiment was 200 g. The liquid product for this study was obtained for the same process parameters with solid-to-liquid ratios of 1:10 and 2:10. SE2 and SE3 samples utilized in the present study did not present any significant differences in terms of chemical composition after solvolysis upon NMR analysis (Supplementary Information), as the most dominant peaks observed were the EG-NMP solvent and derivatives of bisphenol-A, phthalic and methyl groups, thus it is expected to results to similar carbon based nanostructures in terms of morphology, despite the fact that more solvent was used for SE3. In order to ensure repeatability, all samples were tested.

### 2.2. Chemical Vapor Deposition Setup

A horizontal chemical vapor deposition reactor was utilized, using a supported catalyst approach. The reactor apparatus consisted of a gas and liquid injection system, a reaction chamber located inside a high-temperature cylindrical furnace, and a waste gas outlet system. A Thermconcept 50/600/12-3z high-temperature cylindrical furnace (Bremen, Germany) was used. Inside the furnace, quartz or stainless steel tubes with an internal diameter of 34 mm and a length of 1 m and 1.10 m, respectively, were used as reaction chambers, depending on the system pressure. For the growth of nanomaterials, an inert carrier gas (Ar) under steady flow was used. The CVD process can also be carried out by using nitrogen (N_2_) as the inert/carrier gas. However, Ar is suggested in order to avoid the formation of carbon nitrides. In order to introduce the liquid precursors in the system, the liquid was initially placed in a syringe, and it was injected by hand which resulted in an unstable and error-prone injection of the precursor. To overcome this, a custom-made injection system was designed and used as the inlet of the system. More specifically, the syringe was paired with an automated Ugo Basile KMS 100 injection pump (Gemonio, Italy), making the process automatic, offering better stability. The injection system was connected with an extended inlet, carrying the liquid precursor to the inner, high temperature zone of the furnace, ensuring precursor evaporation. The carrier gas directed the gas mixture into the reaction chamber, where it came into contact with the catalytic particles that were placed on the inert substrate (Si wafer).

The catalytic particles were prepared in-house using a precipitation/wet impregnation method, consistent with our previously reported protocols [22,23]. Briefly, zeolite Y (Alfa Aesar, Ward Hill, MA, USA; particle size ~1 μm; specific surface area 975 m^2^/g) was used as the support material. An aqueous solution of Fe(NO_3_)_3_·9H_2_O (Sigma Aldrich, St. Louis, MO, USA) was added in appropriate amounts to achieve a final Fe loading of 20 wt%. The slurry was continuously stirred until near-complete solvent evaporation, followed by drying at 120 °C for 4 h. The material was then calcined at 550 °C under nitrogen flow for 1 h. The catalyst was deposited onto n-type, P-doped Si (100) wafers (dimensions 3.4 × 10 cm) prior to insertion into the reactor. For each experiment, 0.3–0.8 g of catalyst was used, depending on the experimental conditions.

**Figure 1:**
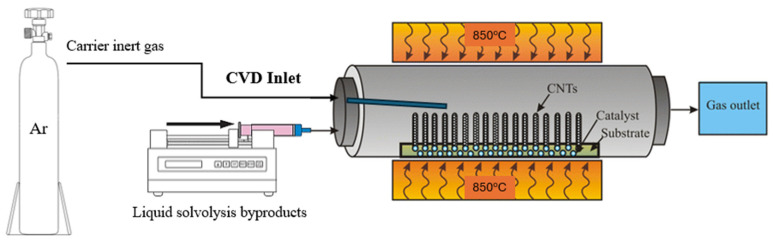
The CVD setup used in the experiments with a redesigned inlet for the automatic injection of liquid precursors.

As the last step, the reaction and growth of the nanomaterials took place, while the exhaust gases were directed to the outlet in a fume hood. The whole system is shown in Figure 1.

### 2.3. Synthesis Protocol

A series of experiments for carbon nanomaterial synthesis through catalytic CVD was conducted, following the supported catalyst approach. The catalyst consisted of iron nanoparticles, impregnated in Y-type zeolite powder. A 425 μm sieve was utilized to sprinkle the catalyst powder over a Si wafer, as inert substrate. The wafer was inserted in the reaction chamber and positioned at the center of the active zone of the furnace. The reactor was tightly sealed, purged with Ar flow for 15 min, and then the heating elements were turned on until the furnace reached the desired temperature. Then the feeding of the precursor was initiated, with an injection rate such as the reaction was to last for 1 h and terminated after that time. Temperature, pressure, catalyst, inert gas, gas flow and reaction time, were kept constant throughout the whole experimental campaign. The temperature and inert gas flow were determined based on previous published research. Parameter values are presented in Table 2.

### 2.4. Characterization Techniques

Upon conclusion of the nanomaterial synthesis campaign, the collected products were subjected to scanning electron microscopy (SEM), transmission electron microscopy (TEM), Raman spectroscopy and TGA for qualitative characterization of the structures produced.

Using SEM (FEI inspect microscope (Hillsboro, OR, USA) with Tungsten filament operating at 25 KeV), the morphology of the carbon nanostructures was imaged, extracting information about the type of nanomaterials developed, the diameter of the structures, and their spatial arrangement. In addition, digital image processing using ImageJ 1.54g software was used to measure the diameters of multiple one-dimensional structures (approx. 100 per sample) from SEM images from many different areas of the sample, and the corresponding statistical sizes were calculated. TEM analysis was carried out via a Jeol 2100 HR, 200 kV, Tokyo, Japan. The samples were dispersed in ethanol prior to their measurement through ultrasonication.

Raman spectroscopy (Renishaw inVia™, Ann Arbor, MI, USA, Reflex micro-Raman system equipped with a 532 nm laser, diffraction grating grid of 1800 L/mm, confocality of 65 µm using a ×20 objective and 5% laser power) was used to collect information on the quality of the structures and to compare the structures produced in each experiment. A 532 nm laser was used. The aim was to identify the characteristic G (~1582 cm^−1^) and D (1350 cm^−1^) peaks. The G peak is related to the carbon–carbon bonds in the graphite structure, while the D peak is related to the defects in the graphite lattice. The intensity ratio between the two peaks (I_D_/I_G_) can provide information about the density of imperfections and the quality of the structures under study [24].

Finally, thermogravimetric analysis (TGA, Netzsch STA 449 Jupiter, Selb, Germany) was performed on selected samples to obtain information about the purity and thermal stability of the materials. The measurements were taken in air (O_2_-N_2_: 80-20%) up to a temperature of 800 °C.

## 3. Results and Discussion

### 3.1. Morphological Analysis

**Figure 2:**
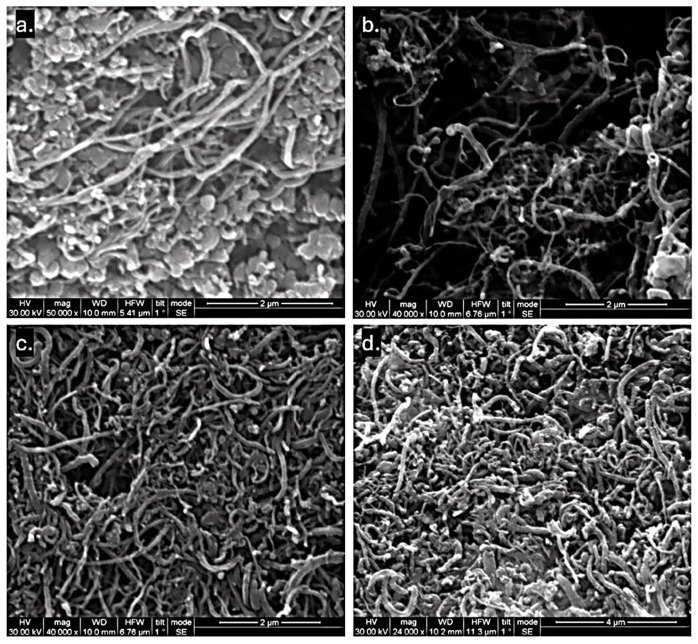
SEM images of nanostructures produced from the following precursors: (**a**) SB1; (**b**) SE1; (**c**) SE2; (**d**) SE3.

SEM was initially employed because it enables rapid morphological characterization and allows statistical analysis over a large number of structures, particularly for CNTs. As shown in Figure 2, the produced samples generally exhibited a mixture of clearly defined long tubular structures (with a length over 2 μm) and amorphous carbon regions. The tubular structures possessed diameters in the nanometer scale, suggesting the presence of Multi-Walled Carbon Nanotubes (MWCNTs) or carbon nanofibers (CNFs) of high aspect ratio.

Table 3 presents the statistical analysis of the nanostructure diameters, indicating average diameters below 100 nm. This value is regarded as a threshold, below which nanostructures are more likely to be MWCNTs, whereas diameters exceeding 100 nm are typically associated with CNFs [25].

The epoxy-derived sample SE1 exhibited the finest morphology, with the lowest average diameter of 57.3 nm and the narrowest standard deviation of 25.5 nm. Furthermore, SE1 showed the highest uniformity, with 92.4% of the measured structures having a diameter of less than 100 nm. Sample SE2 displayed similar characteristics, with an average diameter of 66.2 nm and 91.6% of structures falling below the 100 nm threshold. In contrast, sample SE3 yielded thicker structures, with the highest average diameter of 95.1 nm and a significantly larger interquartile range (IQR) of 58.4 nm, indicating a broader size distribution. Notably, 43.0% of the structures in the SE3 sample exceeded 100 nm in diameter. The polyester-derived sample (SB1) showed intermediate characteristics, with an average diameter of 67.9 nm and 86.6% of structures measuring under 100 nm. However, regions with amorphous carbon were in excess.

**Figure 3:**
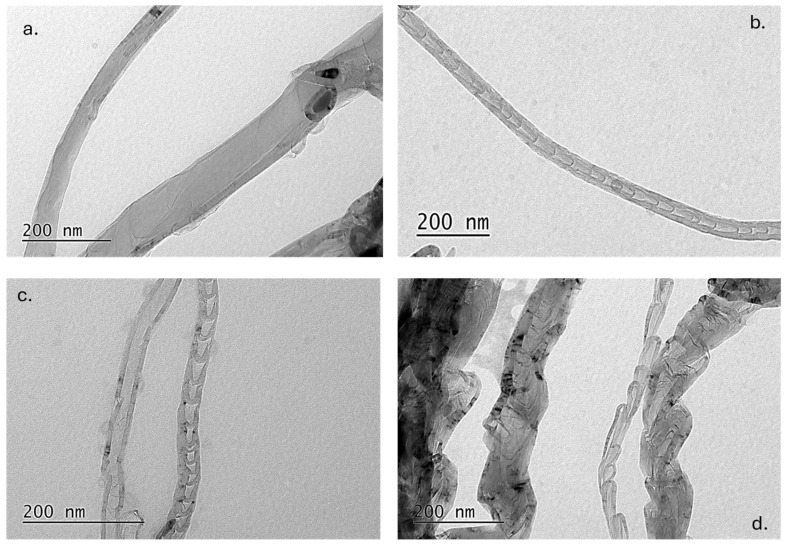
TEM images of nanostructures produced from the following precursors: (**a**) SB1; (**b**) SE1; (**c**) SE2; (**d**) SE3.

It is commonly reported that structures with diameters above ~100 nm are predominantly CNFs, while multi-walled CNTs typically exhibit diameters below this threshold [26,27]. However, the diameter alone is not sufficient for definitive classification, since CNFs may also present diameters below 100 nm. To address this point, TEM analysis was carried out to directly examine the internal structure and wall morphology of the synthesized nanomaterials. TEM enables clear observation of the presence (or absence) of a hollow core, the degree of graphitic ordering, and the arrangement of graphene layers—allowing reliable differentiation between tubular CNT structures and the stacked cone/plate morphology characteristic of CNFs. In Figure 3, representative images from TEM are presented. It is clear that in all cases CNFs exist: In Figure 3a, fibrous structures with filled core are evident (without hollow core); in Figure 3b,c stacked-cup structures are identified, while in Figure 3d, bamboo-like CNFs and distorted fibrous structures can be seen. In the case of SE2 (Figure 3c), MWCNTs are also evident, with similar diameter to the CNFs identified. MWCNTs also co-exist in the rest of the samples, as can be seen in the representative images in Supplementary Information.

TEM analysis also allows for a clear visualization of individual nanostructures in an isolated state, eliminating ambiguity related to bundling, while SEM measurements may include bundled structures, which can lead to overestimation of the actual diameter. For each sample, a statistical analysis based on twenty images and individual measurements was conducted and presented in Table 4. Measurements through ImageJ were performed on the TEM images to obtain more accurate diameter values and compare the SEM-derived apparent diameters with the TEM-derived intrinsic nanotube diameters. From the results it can be concluded that the majority of the CNFs (and CNTs) have diameters below 100 nm (over 85% in all cases). Only a few fibrous 1D structures have diameters over 100 nm. This result seems controversial with the SEM diameter analysis; however, it should be noted that TEM can only examine a very small part of the sample. Thus, the number of fibers that are presented in each image is limited. In the case of SB1 sample, the results of SEM and TEM statistical analyses converged regarding the average diameter calculated. In the case of SE1, TEM analysis resulted in a higher diameter range, while for SE2 and SE3, it was proven that the SEM analysis overestimated the real CNF diameter. To be more realistic, the median diameter should be considered, since the average diameter is affected by thicker structures that scarcely exist in the sample. As a general observation, TEM measurements confirm SEM results in all cases except SE3 sample, which presented the greatest standard deviation concerning the measured average diameters of the produced structures.

### 3.2. Structural Quality Assessment

**Figure 4:**
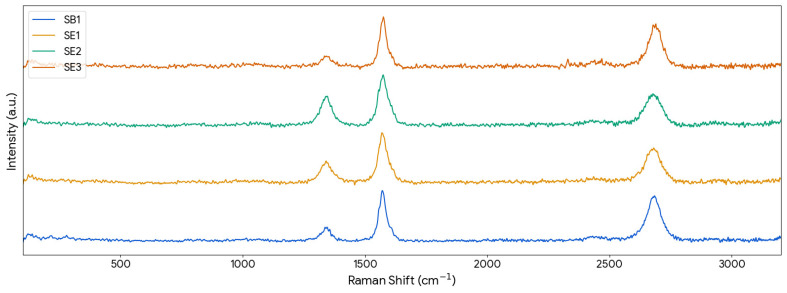
Raman spectra of produced carbon nanostructures.

Raman spectroscopy was utilized to assess the structural quality and graphitic order of the synthesized materials. Raman spectroscopy was further analyzed to support structural identification. CNTs typically exhibit a lower D/G intensity ratio, indicating fewer structural defects and higher graphitic order, as well as a distinct G′ (2D) band associated with well-ordered sp^2^ carbon systems. In contrast, CNFs generally display broader G bands and higher D/G ratios due to their lower structural order. Figure 4 illustrates the Raman spectra for all samples, clearly displaying the characteristic D band (~1350 cm^−1^) and G band (~1582 cm^−1^).

The quantitative data derived from these Raman spectra are summarized in Table 5. The ratio of the D-band to G-band intensity (I_D_/I_G_) was calculated to evaluate the defect density within the carbon lattice. The polyester-derived sample SB1 and the epoxy-derived sample SE3 exhibited the lowest I_D_/I_G_ ratios of 0.28 and 0.25, respectively. This indicates a relatively higher degree of graphitization and fewer structural defects compared to the other samples. Conversely, samples SE1 and SE2 showed higher I_D_/I_G_ ratios of 0.48 and 0.58, respectively, suggesting a higher prevalence of imperfections in these structures. Nevertheless, these values remain relatively low and are comparable to, or even lower than, those commonly reported in the literature for MWCNTs synthesized from virgin precursors, ranging from 0.2 to 1.3 [28,29,30,31,32,33,34,35,36,37].

A correlation between the MWCNT diameter and the I_D_/I_G_ ratio has been reported in the literature, with smaller-diameter nanotubes typically exhibiting higher I_D_/I_G_ values [28]. This behavior is attributed to the increased curvature of the graphene layers in smaller nanotubes, which induces lattice strain and increases the likelihood of defects, thereby enhancing the D-band intensity. This trend is corroborated in the present study, as the I_D_/I_G_ ratios closely follow the average diameter trends of the samples.

### 3.3. Thermal Analysis

**Figure 5:**
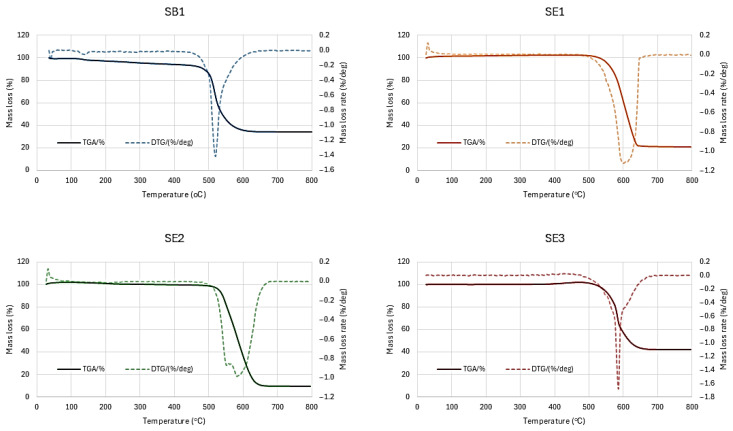
Thermogravimetric analysis results of produced carbon nanostructures.

The thermal stability and purity of the carbon nanomaterials were evaluated via TGA carried out in air atmosphere. The resulting TGA curves are presented in Figure 5, and the extracted thermal parameters are detailed in Table 6.

All samples demonstrate similar thermal profiles, starting with a mild-to-zero mass loss region from 30 to 400 °C, where volatile compounds like water are evaporated and amorphous carbon is decomposed. Following that a steep mass loss curve occurs where the graphitic carbon nanomaterials are oxidized and released as gas phase products, causing a considerable mass loss at the 500–650 °C range, typical with CNTs and CNFs decomposition behavior. The temperature at which the mass loss rate is maximized is considered as the oxidation temperature of the carbon nanomaterials. At the highest temperature range over 650 °C, the material remains relatively stable, leaving a solid residue attributed to the iron and zeolite from the catalyst [38]. Nanomaterial purity can be defined by subtracting the initial mass loss and final residue from the total mass.

Sample SE2 demonstrated the highest purity among the tested materials, achieving a carbon purity of 90.3% with a residual mass of only 9.2% at 800 °C. The oxidation temperature for SE2 was recorded at 576.2 °C. Sample SE1 also showed high thermal stability with the highest oxidation temperature of 596.1 °C and a purity of 79.0%.

Samples SB1 and SE3 exhibited lower purity levels. SB1 had a purity of 59.9% and a residue of 34.2%, while it presented the lowest thermal stability The relatively steeper initial mass loss phase of 5.9% before 400 °C, indicated high presence of amorphous carbon, as also proved in SEM analysis, in combination with high catalyst content, resulting in degraded nanomaterial purity. Sample SE3 showed the lowest purity at 57.9% and the highest residual mass of 42.1%.

It should be noted that the width of the DTA curves follow the same trend as the Raman results, since samples SB1 and SE3 that presented the lowest I_D_/I_G_ ratio, had also the narrowest DTA curves. This indicates that these samples showed high uniformity in terms of the structures that have been formed.

## 4. Conclusions

The results presented in the current study validate the hypothesis that polymer recycling liquid waste can serve as an effective carbon source for the growth of carbon nanomaterials. By utilizing these complex, carbon-rich mixtures of monomers and oligomers, the process addresses the urgent need for high-value applications that enhance the economic viability of thermoset recycling methods. The experimental results demonstrate that these waste precursors can successfully produce 1D nanostructures (MWCNTs and CNFs). Epoxy-derived solvolysis by-products yielded high quality carbon nanomaterials, demonstrating the versatility and reliability of CVD. For the polyester-derived solvolysis by-product, it was proven that 1D carbon nanostructures can also be produced, however further optimization is needed, mainly on the synthesis parameters, in order to obtain high quality and uniform MWCNTs. Within the investigated range (20 wt% Fe loading and 0.3–0.8 g total catalyst mass), no significant change in product morphology or purity was observed. However, catalyst amount influences the overall yield due to the increased number of active growth sites. To further advance this methodology, the systematic optimization of CVD growth parameters (reaction temperature, reaction duration) and reactor set up will be essential, while a transition toward continuous synthesis through a vertical CVD system will enhance scalability, considering a floating catalyst approach instead of a supported catalyst on a defined substrate. This will enable the continuous introduction of catalyst simultaneously with the carbon feedstock, in order to avoid deactivation phenomena of the catalyst due to possible impurities of the liquid byproducts. In this way, a sustainable process for the valorization of solvolysis wastes is suggested for the production of high-added value carbon based nanomaterials.

## Figures and Tables

**Table 1:** Precursors origin.

Sample	SB1	SE1	SE2	SE3
**Process**	Solvolysis	Solvolysis	Solvolysis	Solvolysis
**Polymer**	Unsaturated polyester	Epoxy resin	Epoxy resin	Epoxy resin
**Conditions**	200 °C/4–5.5 h	Various (Mix)	190 °C/3 h	191 °C/3 h
**Solvent**	PEG	Various (Mix)	EG/NMP 1:1 mol	EG/NMP 1:1 mol
**Catalyst**	NaOH 5 g/kg	Unknown	TBD 0.025 mol/L	TBD 0.025 mol/L
**Material/solvent ratio (*w*/*w*)**	1:20	Unknown	2:10	1:10

**Table 2:** Experimental parameters.

Parameter	Value
Catalyst:	Fe nanoparticles on zeolite substrate
Reaction temperature (T_r_):	850 °C
Reaction time (t_r_):	1 h
Carrier inert gas:	Argon
$\dot{V}$ of inert gas during reaction:	260 mL/min
$\dot{V}$ of inert gas during purging:	190 mL/min

**Table 3:** Statistical analysis of nanostructures diameter distribution through SEM.

Sample	Average d (nm)	St. Dev. (nm)	IQR (nm)	Median d (nm)	Percentage < 100 nm (%)	Percentage > 100 nm (%)
SB1	67.9	25.2	34.3	61.6	86.6	13.4
SE1	57.3	25.5	24.2	52.0	92.4	7.6
SE2	66.2	24.5	27.3	62.4	91.6	8.4
SE3	95.1	39.0	58.4	85.1	57.0	43.0

**Table 4:** Statistical analysis of nanostructures diameter distribution through TEM.

Sample	Average d (nm)	St. Dev. (nm)	IQR (nm)	Median d (nm)	Percentage < 100 nm (%)	Percentage > 100 nm (%)
SB1	65.40	34.75	36.58	61.89	0.90	0.10
SE1	72.08	49.24	39.28	57.51	0.86	0.14
SE2	56.92	23.07	35.52	52.90	0.96	0.04
SE3	69.13	34.61	37.30	60.45	0.85	0.15

**Table 5:** Raman spectroscopy data.

Sample	Peak (cm^−1^)	Intensity (AU)	FWHM Bandwidth (cm^−1^)	I_D_/I_G_
SB1	D	145.66	40.4	0.28
	G	513.63	35.7	
SE1	D	177.76	58.8	0.48
	G	399.26	44.5	
SE2	D	228.94	53.9	0.58
	G	391.86	49.7	
SE3	D	72.144	58.0	0.25
	G	306.47	35.5	

**Table 6:** TGA data.

Sample	SB1	SE1	SE2	SE3
Mass loss up to 400 °C (%)	5.9	0.0	0.6	0.0
Residue at 800 °C (%)	34.2	20.97	9.2	42.1
Oxidation temperature (°C)	514.5	596.1	576.2	581.1
Purity (%)	59.9	79.0	90.3	57.9

## Data Availability

The data presented in this study are openly available in EuReComp: Results and Deliverables collection at doi:10.5281/zenodo.18449291, reference number: 10.5281/zenodo.18449292 (accessed on 16 March 2026).

## Supplementary Materials

The following supporting information can be downloaded at https://www.mdpi.com/article/10.3390/ma19061266/s1, Figure S1: ^1^H NMR spectra of blank EG-NMP solvent with TBD. Figure S2: ^1^H NMR spectra of liquid product after WTB solvolysis at 2:10 ratio in EG-NMP solvent with 0.025 mol TBD. Figure S3: ^1^H NMR spectra of liquid product after WTB solvolysis at 1:10 ratio in EG-NMP solvent with 0.025 mol TBD.

## Abbreviations

The following abbreviations are used in this manuscript and presented in alphabetical order:

CVD	Chemical Vapor Deposition
CNFs	Carbon Nanofibers
CNTs	Carbon Nanotubes
EG	Ethylene Glycol
FWHM	Full Width at Half Maximum
GFRP	Glass Fiber-Reinforced Polymer
IQR	Interquartile Range
MWCNTs	Multi-Walled Carbon Nanotubes
NMP	1-methyl-2-pyrrolidinone
NMR	Nuclear Magnetic Resonance
PEG	Polyethylene Glycol
SEM	Scanning Electron Microscopy
TBD	1,5,7-Triazabicyklo[4.4.0]dek-5-en
TGA	Thermogravimetric Analysis
$\dot{V}$	Volumetric flow rate
WTB	Wind Turbine Blades
