# Supplementary material for: Circular Approach to Composite Materials: Synthesis of Carbon Nanomaterials from Polymer Recycling Liquid By-Products
Source: Materials (Basel). 2026 Mar 23;19(6):1266. doi: 10.3390/ma19061266 (PMC13028497; doi:10.3390/ma19061266)

Article

Circular Approach to Composite Materials: Synthesis of Carbon Nanomaterials from Polymer Recycling Liquid By-Products

Evangelos Tsimis [1], Stefania Termine [1], Maria Modestou [1], Aikaterini-Flora Trompeta [1,*], Szymon Sobek [2], Marcin Sajdak [3], Jakub Adamek [4], Sebastian Werle [5] and Costas Charitidis [1,*]

[1] Research Lab of Advanced, Composite, Nano-Materials and Nanotechnology (R-NanoLab), Materials Science and Engineering Department, School of Chemical Engineering, National Technical University of Athens, 9 Heroon Polytechniou, 15773 Athens, Greece; etsimis@chemeng.ntua.gr (E.T.); stermine@chemeng.ntua.gr (S.T.)
[2] Department of Heating, Ventilation and Dust Removal Technology, Faculty of Energy and Environmental Engineering, Silesian University of Technology, Stanisława Konarskiego 20, 44-100 Gliwice, Poland; szymon.sobek@polsl.pl
[3] Department of Air Protection, Faculty of Energy and Environmental Engineering, Silesian University of Technology, Stanisława Konarskiego 22B, 44-100 Gliwice, Poland; marcin.sajdak@polsl.pl
[4] Department of Organic Chemistry, Bioorganic Chemistry and Biotechnology, Faculty of Chemistry, Silesian University of Technology, B. Krzywoustego 8, 44-100 Gliwice, Poland; jakub.adamek@polsl.pl
[5] Department of Thermal Technology, Faculty of Energy and Environmental Engineering, Silesian University of Technology, Stanisława Konarskiego 22, 44-100 Gliwice, Poland; sebastian.werle@polsl.pl
* Correspondence: ktrompeta@chemeng.ntua.gr (A.-F.T.); charitidis@chemeng.ntua.gr (C.C.)

EoL Composites chemical analysis

The EoL GFRPs coming from WTB wastes were analyzed prior their recycling through solvolysis. Ultimate and proximate analyses with ash composition were carried out using a plasma spectrometer, the Thermo iCAP 6500 Duo ICP. All analyses were conducted using standards and procedures, including the following: the moisture content was determined based on thermogravimetric method according to PN-G-04560:1998; the gravimetric determination of volatile matter and ash contents was done according to EN 15402:2011 and EN ISO 21656:2021–06; and ultimate analyses were done according to EN ISO 21663:2021–06 and high-temperature combustion with IR detection. Following the EN ISO 16993:2016–09 standard, the $O_{a.d.}$ was determined as the difference.

The results of the samples composition are: moisture (M_a) 1.3 wt.%, volatile matter (VM) 58.7 wt.%, ash content (A_a) 41.2 wt.%, carbon (C_a) 69.7 wt.%,

oxygen ($O_{a.d.}$) 19.8 wt.%, hydrogen (H_d) 7.3 wt.%, nitrogen (N_a) 3.0 wt.%, and sulfur (S_a) <0.5 wt.%

Epoxy-based Liquid by-product characterization through NMR

For the assessment of the solvolysis liquid product composition, the NMR analysis was used. The ^{1}H spectra were recorded in DMSO-d6 on an Agilent Magnet 400 spectrometer at operating frequencies of 400 MHz. For the one-time measurement, the 200µl of sample was added to the 500 µl of DMSO-d6 and used, while for the quantitative analysis, the standard (dimethyldiphenylsilane) was implemented. During analysis, the temperature was maintained at 25 °C.

For this study, the three delivered liquid product samples did not present any significant differences in terms of chemical composition after solvolysis. The most dominant peaks observed during NMR were the EG-NMP solvent and derivatives of bisphenol-A, phthalic and methyl groups. Detailed NMR spectra are presented in the following:

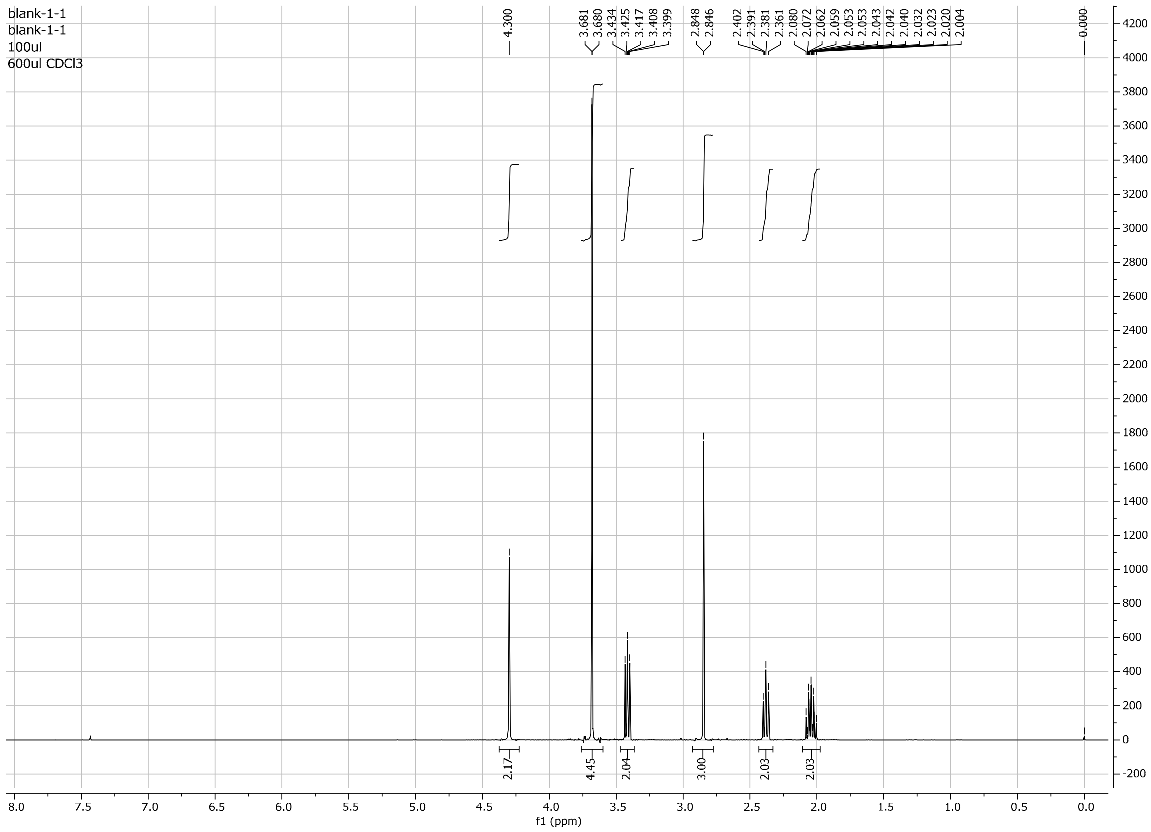

Figure S1. ^{1}H NMR spectra of blank EG-NMP solvent with TBD.

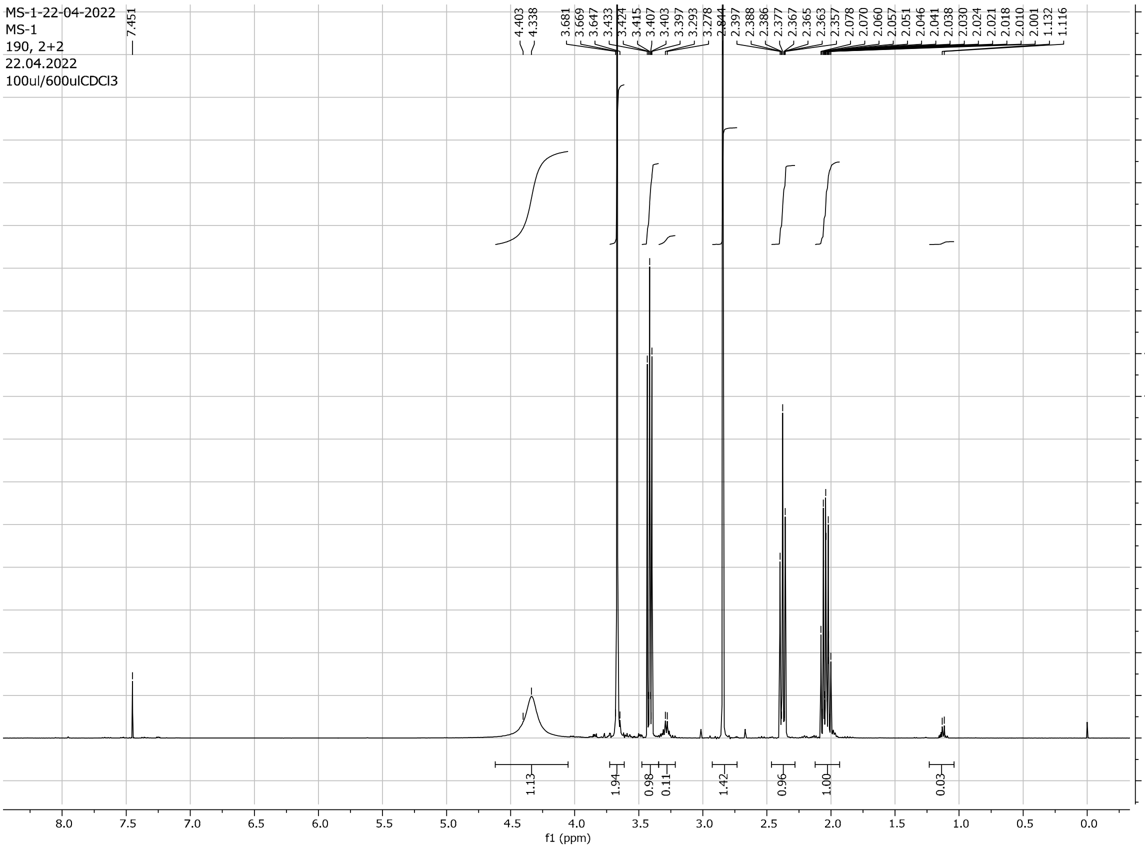

Figure S2. ^{1}H NMR spectra of liquid product after WTB solvolysis at 2:10 ratio in EG-NMP solvent with 0.025 mol TBD.

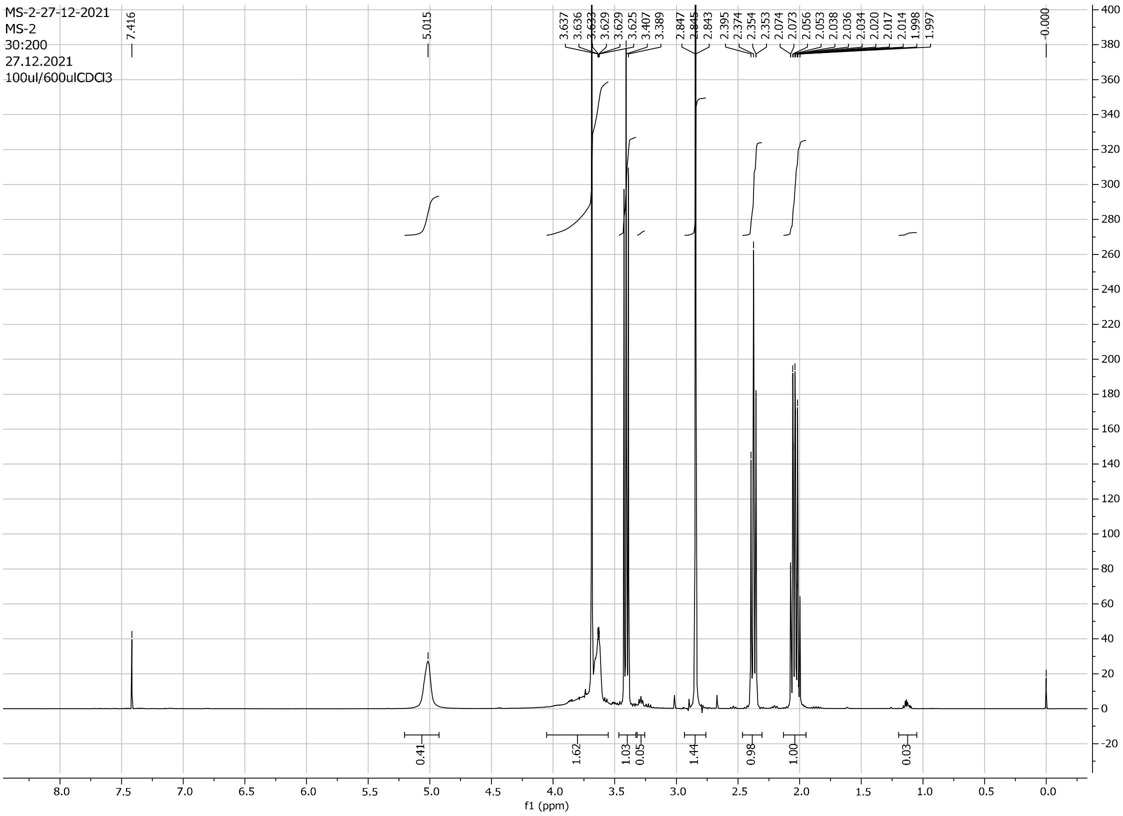

Figure S3. ^{1}H NMR spectra of liquid product after WTB solvolysis at 1:10 ratio in EG-NMP solvent with 0.025 mol TBD.

Polyester-based Liquid by-products characterization through ICP-OES

The characterization and subsequent treatment of these liquid wastes were conducted to evaluate their chemical composition. Inductively coupled plasma optical emission spectroscopy (ICP-OES) was employed to quantify dissolved elemental species, with particular focus on sodium and silicon concentrations derived from matrix degradation and partial fiber dissolution. The analysis showed significant concentrations of dissolved species, notably sodium (780.7 mg/L) and silicon (138.62 mg/L), indicative of the alkaline solvolytic environment mediating both matrix depolymerization and a partial attack on glass fiber surfaces at high NaOH loadings. Additionally, the presence of aluminum (87.14 mg/L) further supports the dissolution of the silicate–aluminate network in the glass fibers, while trace amounts of other elements such as titanium and zinc suggest the influence of protective coatings, which were less affected by the solvolysis process.

Representative TEM Images

Sample SB1

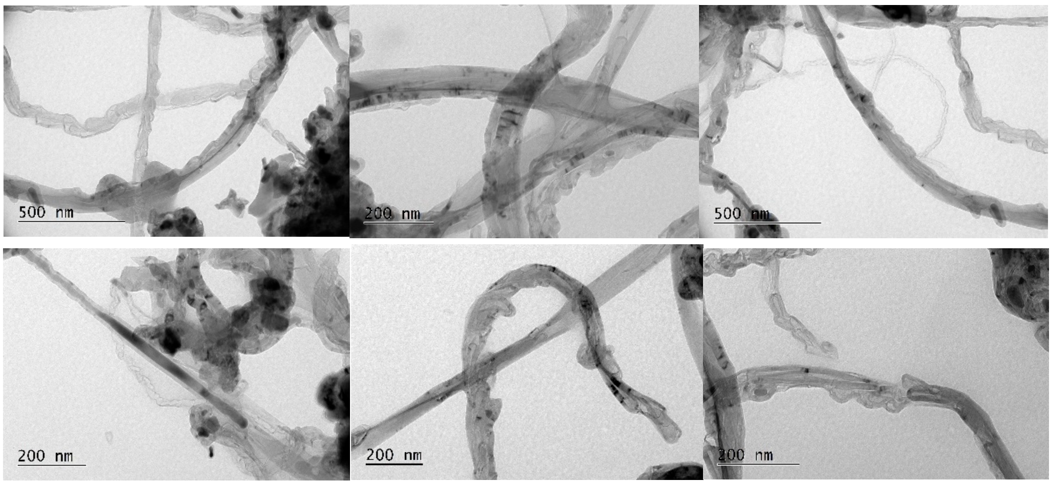

Sample SE1

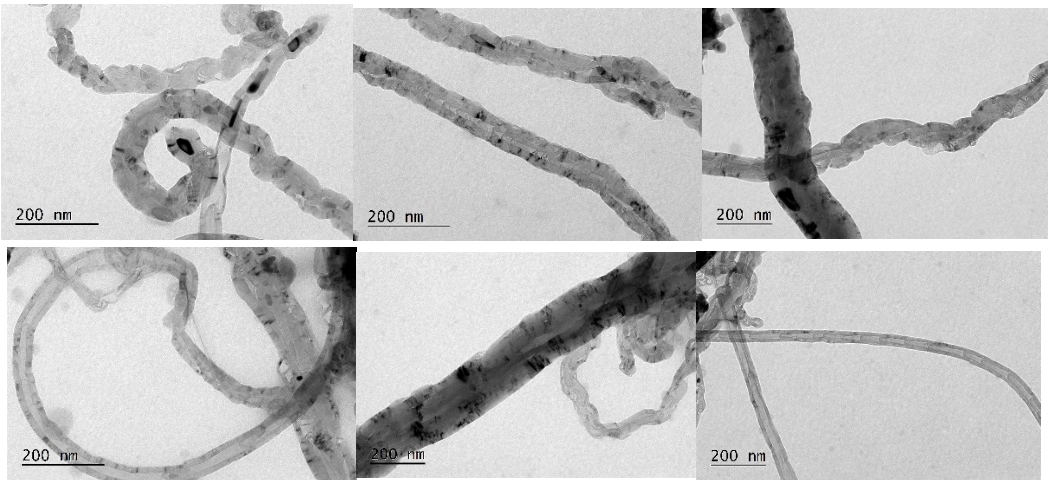

Sample SE2

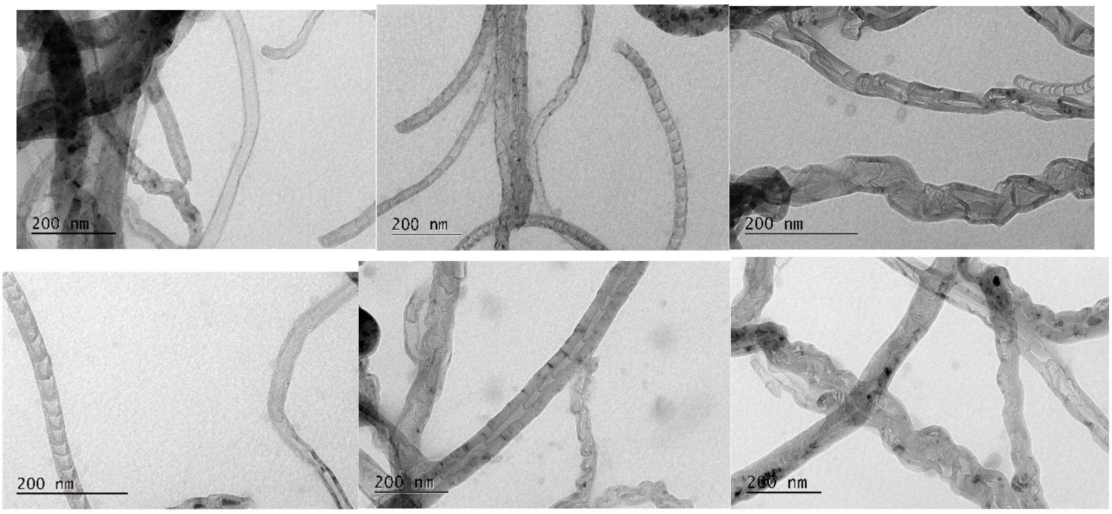

Sample SE3

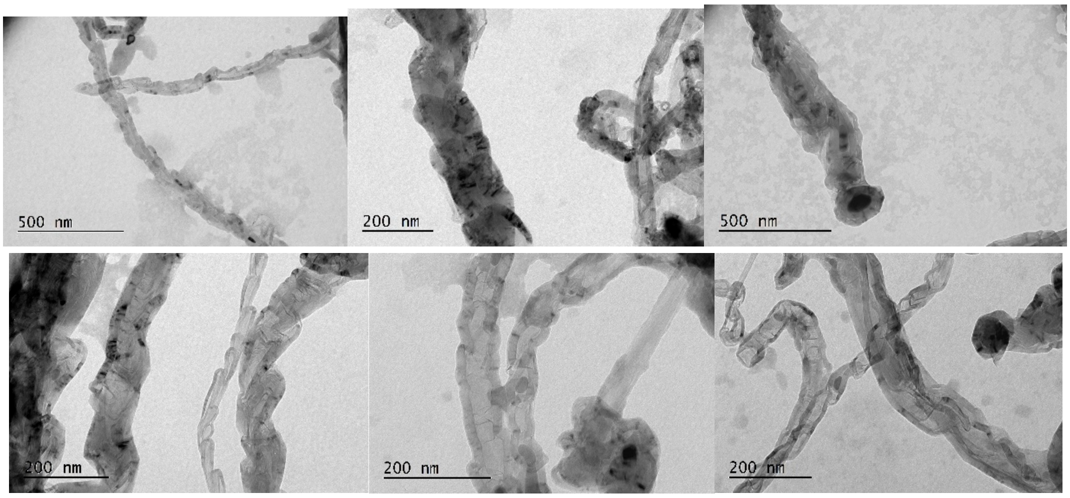

Supplement: Supplementary file 1 [file materials-19-01266-s001.zip › materials-4157422-supplementary.pdf]
